# Oral Health Impact Profile and Associated Variables in Southern Brazilian Drug Users

**Published:** 2018-10

**Authors:** Susan Jaccoud Ribeiro DE SOUZA, Andrea de Castro DOS SANTOS, Milena Binhame ALBINI, Marilisa Carneiro Leão GABARDO, Antonio Adilson Soares DE LIMA, Maria Ângela Naval MACHADO

**Affiliations:** 1. Dept. of Dentistry, Universidade Federal do Paraná, Curitiba, Paraná, Brazil; 2. School of Life Sciences, Universidade Positivo, Curitiba, Paraná, Brazil

**Keywords:** Oral health, Self-perception, Quality of life, Substance-related disorders

## Abstract

**Background:**

This study investigated the association between sociodemographic, behavioral, oral health, variables of drug consumption, and the short version of the Oral Health Impact Profile (OHIP-14), in Southern Brazilian drug users.

**Methods:**

In this cross-sectional study, 202 drug users aged 18 or over admitted for treatment at the Institute for Research and Treatment of Alcoholism (*Instituto de Pesquisa e Tratamento do Alcoolismo* -IPTA) from the municipality of Campo Largo, PR, Brazil, from 2012 to 2014, were involved. They answered a questionnaire and were examined by a calibrated researcher. Data were collected and bivariate (Chi-square test) and multivariate (logistic regression and Wald’s test) analyses were performed.

**Results:**

There was statistical difference (*P*<0.05) in bivariate analysis and the worst impacts were reported by non-whites, those who reported feeling metallic taste in mouth and tooth mobility, which use cigarettes for over 15 yr and consume more than 3 g of crack/day, with DMFT >10, and number of teeth in mouth ≤27. In the multivariate analysis, statistical difference remained, except for ethnicity (*P*=0.207).

**Conclusion:**

Self-perception of oral health was associated with the variables explored, which suggests the need for strategies focused on this population.

## Introduction

Drug dependence is defined as “a state psychic and sometimes also physical, resulting from the interaction between a living organism and a drug, characterized by behavioral and others responses that always include a compulsion to take the drug on a continuous or periodic basis in order to experience its psychic effects, and sometimes to avoid the discomfort of its absence. Tolerance may or may not be present. A person may be dependent on more than one drug” (1). Drug consumption is considered a public health problem and is associated not only with health problems, but also the social, such as violence, accidents, and death (2). At the global level, approximately 275 million people have used some illicit substance in 2016 (2). The most commonly used is marijuana, followed by cocaine and opioids (2). In Brazil, there is an estimated one million illicit drug users (DU) (other than marijuana). Of these, 370000 (35.0%) consume crack and cocaine derivatives (paste-based, merla and oxy), including 50000 children and adolescents (3).

Illicit substances cause oral health damage and remain even after the use has stopped. The reported oral problems include xerostomia (4), high number of decayed, missing and filled (DMFT) (4–8) reduced saliva buffering capacity (5), tooth loss (6), bruxism (4,6), and periodontal disease (4,6–9).

In addition, for DU, the effect of drugs alone on oral diseases is less than the social and behavioral effects (9,10). The addiction seems to interfere with quality of life because it is directly related to the imbalance between the combination of psychological well-being and physical health (11).

In general health, self-perception seems to depend on sociodemographic factors and the environmental context in which individuals live (12). In oral health is important to seek the association between health-disease process and its interference in daily activities of individuals that can impact on quality of life (13). In addition to clinical measures, perceived health status, and the account of the physical, social and psychological functions are seen as independent variables, but correlated, constituting the oral health-related quality of life (14).

Individuals with low socioeconomic status and the ones which have tooth loss often have greater impact of oral health on quality of life (l5). However, these data related to the DU is scarce (9,16). Thus, the present study aimed to evaluate the variables associated with the self-perceived impact of oral health on quality of life in DU in recovery at the Institute for Research and Treatment of Alcoholism (*Instituto de Pesquisa e Tratamento do Alcoolismo* – IPTA), in the municipality of Campo Largo, PR, Brazil.

## Methods

The Ethics Committee in Research of the Universidade Federal do Paraná (number: CEP/SD 1125.050.11.05) approved this study. Participants signed the Informed Consent Statement.

In this cross-sectional study, a non-probabilistic sample was obtained from 2012 to 2014. Those included were crack and/or marijuana smokers, and also cocaine users or not. They answered a questionnaire about personal behavioral data, and the Oral Health Impact Profile, in short-form, the OHIP-14 (17). A trained and calibrated researcher (kappa=0.82) performed the collection. The IPTA admits male patients, aged at least 18 yr, inserted into a program for treatment and prevention during 45 with a multi-professional team.

The variables obtained were: Sociodemographic - age (in years), ethnicity (white, brown, black, native, and asian), marital status (married, single, divorced, separated, widowed), studying (yes, no), working (yes, no), if have children (yes, no), schooling (incomplete primary education, complete primary education, incomplete secondary education, secondary education, incomplete higher education, university degree, technical course), living alone (yes, no); Behavioral - brushing teeth (yes, no) and frequency (times/day), flossing (yes, no), use of toothpaste (yes, no); Oral health - feeling metallic taste (yes, no), feeling tooth mobility (yes, no), visit to the dentist in the past six months (yes, no), decayed, missing and filled teeth (DMFT) (18), and number of teeth present; Drug use - type(s) (cigarette, marijuana, crack cocaine*,* cocaine, lysergic acid diethylamide (LSD), ecstasy or oxy) (yes, no), how long they use the drug (in years), and daily consumption.

Given the distribution of variables, ethnicity was classified as white and non-white; marital status is married and unmarried; schooling in high (technical course, high school graduate, incomplete university degree and university degree); medium (complete primary education and incomplete high school) and low (no schooling and incomplete primary education); and frequency of brushing in ≥ 3 and ≤ 2 times/day.

They answered the OHIP-14, validated for the Brazilian context (19). This instrument was applied as an interview for the period of 12 months prior to admission. The guiding question was: “How frequently did the following occur during the last six months because of problems with your teeth, your mouth or dentures?” The questions were answered according to a coded scale: 0- never; 1- rarely; 2- sometimes; 3- constantly/often and 4- always. The higher the value assigned by the respondent, the worse the perception of the problem (20). Each respondent presented 14 replies corresponding to the questions applied. To obtain the final score on the scale, the additive method was used. This procedure allows an assessment in terms of severity (17).

The dichotomization of this variable was based on the median of: OHIP-14≤15 (better condition) and OHIP-14>15 (worse condition).

Data were analyzed using the SPSS, version 19.0 (SPSS Inc, Chicago, IL, USA). Quantitative variables were dichotomized by the mean or median, according to the normality test (Kolmogorov-Smirnov). After the descriptive analysis, associations were explored between the explanatory variables and the OHIP-14 (Chi-square test). The variables in the previous step with *P*<0.20 were included in the logistic regression and Wald’s tests. Results were considered statistically significant when *P*<0.05.

## Results

Two hundred and two male DU were evaluated. The mean age was 34 yr old (SD=9.1; min=18, max=62).

The mean DMFT was 11 (SD=6.7). The mean of healthy teeth was 15, and 3.8 decayed, 0.7 filled with caries, 2.9 filled without caries, 2.7 missing due to caries, and 2.8 missing by other reasons (periodontitis/could not inform).

The prevalence of OHIP-14 > 15 was 49.0% (n=99). Tables 1, 2 and 3 show the distribution, prevalence and odds ratio (OR) unadjusted and adjusted to the OHIP-14 according to explanatory variables.

**Table 1:** Distribution, prevalence and odds ratio (OR) unadjusted and adjusted to the OHIP-14 according to socio-demographic variables in a population of DU from Campo Largo municipality, PR, Brazil, 2012–2014

***Variables***	***n (%)***	***OHIP-14*≤*15 n=103 (51.0%)***	***OHIP-14>15 n=99 (49.0%)***	P value^*^	***OR unadjusted (95%IC)***	P value^**^	***OR adjusted (95%IC)***
Age (yr)							
≤34	116 (57.4)	63 (61.2)	53 (53.5)	0.320	1		
> 34	86 (42.6)	40 (38.8)	46 (46.5)		1.36 (0.78–2.39)		
Ethnicity							
White	121 (75.2)	66 (82.5)	55 (67.9)	0.044	1	0.207	1
Non-white	40 (24.8)	14 (17.5)	26 (32.1)		2,22 (1.06–4.67)		2.01 (0.67–6.00)
Marital status							
Married	46 (28.6)	46 (57.5)	39 (48.1)		1		
Not married	115 (71.4)	34 (42.5)	42 (51.9)	0.270	1.45 (0.78–2.71)		
Studying							
Yes	19 (9.5)	13 (12.7)	6 (6.1)	0.147	1		
No	183(90.5)	89 (87.3)	93 (93.9)		2.26 (0.82–6.21)		
Working							
Yes	118 (58.4)	59 (57.3)	59 (59.6)	0.776	1		
No	84 (41.6)	44 (42.7)	40 (40.4)		0.90 (0.51–1.59)		
Children							
Yes	78 (48.4)	35 (43.8)	43 (53.1)		1		
No	83 (51.6)	45 (56.3)	38 (46.9)	0.271	0.68 (0.36–1.27)		
Schooling							
High	35 (21.7)	7 (10.8)	2 (2.9)		1	0.567	1
Medium	54 (33.5)	28 (43.1)	26 (27.1)		3.25 (0.61–17.0)	0.394	0.45 (0.07–2.80)
Low	72 (44.8)	30 (46.2)	41 (60.0)	0.097	4.78 (0.92–24.6)	0.407	0.67 (0.26–1.70)
Living alone							
No	95 (59.0)	47 (58.8)	48 (59.3)		1		
Yes	66 (41.0)	33 (41.3)	33 (40.7)	1.000	0.97 (0.52–1.83)		

For ethnicity, marital status, children, schooling, living alone, n = 161 //Bold values are statistically significant (*P*<0.05) //

* Chi-square test

** Logistic regression and Wald′s test

**Table 2:** Distribution, prevalence and odds ratio (OR) unadjusted and adjusted to the OHIP-14 according to behavioral and oral health variables in a population of DU from Campo Largo municipality, PR, Brazil, 2012–2014

***Variables***	***n (%)***	***OHIP-14*≤*15 n=103 (51.0%)***	***OHIP-14>15 n=99 (49.0%)***	P value^*^	***OR unadjusted (95%IC)***	P value^**^	***OR adjusted (95%IC)***
Brushing the teeth							
Yes	191 (94.6)	99 (96.1)	92 (92.9)	0.366	1		
No	11 (5.4)	4 (3.9)	7 (7.1)		1.88 (0.53–6.64)		
Frequency of brushing (times/day)							
≥3	124 (61.4)	62 (60.2)	62 (62.6)	0.773	1		
≤2	78 (38.6)	41 (39.8)	37 (37.4)		0.90 (0.51–1.59)		
Flossing							
Yes	47 (23.3)	27 (26.2)	20 (20.2)	0.324	1		
No	155 (76.7)	76 (73.8)	79 (79.8)		1.40 (0.72–2.71)		
Use of toothpaste							
Yes	195 (96.5)	101 (98.1)	94 (94.9)	0.272	1		
No	7 (3.5)	2 (1.9)	5 (5.1)		2,68 (0.50–14.18)		
Felling metallic taste							
No	153 (75.7)	88 (85.4)	65 (65.7)	0.002	1	0.003	1
Yes	49 (24.3)	15 (14.6)	34 (34.3)		3.06 (1.54–6.09)		5.66 (1.79–17.89)
Tooth mobility							
No	147 (72.8)	86 (83.5)	61 (61.6)	0.001	1	0.265	1
Yes	55 (27.2)	17 (16.5)	38 (38.4)		3.15 (1.62–6.09)		1.87 (0.62–5.64)
Visit to the dentist							
Yes	183 (90.6)	94 (91.3)	89 (89.9)	0.812	1		
No	19 (9.4)	9 (8.7)	10 (10.1)		1.17 (0.45–3.02)		
DMFT							
≤10	114 (56.4)	68 (66.0)	46 (46.5)	0.007	1	0.027	1
> 10	88 (43.6)	35 (34.0)	53 (53.5)		2.23 (1.26–3.94)		2.93 (1.13–7.59)
Number of teeth in the mouth							
> 27	88 (43.6)	61 (59.2)	27 (27.3)	<0.01	1	0.026	1
≤27	114 (56.4)	42 (40.8)	72 (72.7)		3.87 (2.14–6.99)		3.05 (1.14–8.14)

Bold values are statistically significant (*P*<0.05).

* Chi-square test

** Logistic regression and Wald's test

**Table 3:** Distribution, prevalence and odds ratio (OR) unadjusted and adjusted to the OHIP-14 according to drug consumption variables in a population of DU from Campo Largo municipality, PR, Brazil, 2012–2014

***Variables***	***n (%)***	***OHIP-14*≤*15 n=103 (51.0%)***	***OHIP-14>15 n=99 (49.0%)***	P value^*^	***OR unadjusted (95%IC)***	P value^**^	***OR adjusted (95%IC)***
Cigarette use							
No	27 (13.4)	22 (21.4)	5 (5.1)	**0.001**	1	**0.005**	1
Yes	175 (86.6)	81 (78.6)	94 (94.9)		**5.10 (1.84–14.09)**		**11.22 (2.11–59.71)**
Cigarette use time (in years)							
≤15	102 (50.5)	65 (63.1)	37 (37.4)	**<0.01**	1	0.140	1
> 15	100 (49.5)	38 (36.9)	62 (62.6)		**2.86 (1.61–5.07)**		2.01 (0.79–5.08)
Cigarette amount (number/day)							
≤ 20	174 (86.1)	90 (87.4)	84 (84.8)	0.686	1		
> 20	28 (13.9)	13 (12.6)	15 (15.2)		1.23 (0.55–2.75)		
Alcohol use							
No	41 (20.3)	21 (20.4)	20 (20.2)	1.000	1		
Yes	161 (79.7)	82 (79.6)	79 (79.8)		1.01 (0.50–2.00)		
Alcohol use time (in years)							
≤ 13	102 (50.5)	55 (53.4)	47 (47.5)	0.482	1		
> 13	100 (49.5)	48 (46.6)	52 (52.5)		1.26 (0.72–2.20)		
Alcohol amount (liters/day)							
≤ 1	132 (65.3)	70 (68.0)	62 (62.6)	0.462	1		
> 1	70 (34.7)	33 (32.0)	37 (37.4)		1.26 (0.70–2.26)		
Marijuana use							
No	64 (31.7)	34 (33.0)	30 (30.3)	0.763	1		
Yes	138 (68.3)	69 (67.0)	69 (69.7)		0.98 (0.54–1.79)		
Marijuana use time (in years)							
≤7	102 (50.5)	54 (52.4)	48 (48.5)	0.673	1		
> 7	100 (49.5)	49 (47.6)	51 (51.5)		1.13 (0.62–2.05)		
Marijuana amount (number/day)							
≤2	110 (54.5)	59 (57.3)	51 (51.5)	0.480	1		
> 2	92 (45.5)	44 (42.7)	48 (48.5)		1.26 (0.72–2.19)		
Crack use							
No	0 (0.0)	0 (0.0)	0 (0.0)		1 -		
Yes	202 (100.0)	103 (100.0)	99 (100.0)		0.96 (0.01–48.92)		
Crack use time (in years)							
≤ 7	102 (50.5)	54 (52.4)	48 (48.5)	0.673	1		
> 7	100 (49.5)	49 (47.6)	51 (51.5)		1.17 (0.67–2.03)		
Crack amount (g/day)							
≤3	119 (58.9)	53 (51.5)	66 (66.7)	**0.032**	1	0.389	1
> 3	83 (41.1)	50 (48.5)	33 (33.3)		**0.53 (0.30–0.93)**		**0.66 (0.26–1.67)**
Cocaine use							
No	88 (43.6)	45 (43.7)	43 (43.4)	1.000	1		
Yes	114 (56.4)	58 (56.3)	56 (56.6)		1.01 (0.57–1.76)		
Cocaine use time (in years)							
≤0	138 (68.3)	68 (66.0)	70 (70.7)	0.546	1		
> 0	64 (31.7)	35 (34.0)	29 (29.3)		0.80 (0.44–1.45)		
Cocaine amount (g/day)							
≤0	93 (46.0)	45 (43.7)	48 (48.5)	0.572	1		
> 0	109 (54.0)	58 (56.3)	51 (51.5)		0.82 (0.47–1.43)		
LSD use							
No	184 (91.1)	91 (88.3)	93 (93.9)	0.218	1		
Yes	18 (8.9)	12 (11.7)	6 (6.1)		0.48 (0.17–1.35)		
LSD use time (in years)							
≤0	196 (97.0)	99 (96.1)	97 (98.0)	0.360	1		
> 0	6 (3.0)	4 (3.9)	2 (2.0)		0.51 (0.09–2.88)		
LSD amount (tablets/day)							
≤0	184 (91.1)	91 (88.3)	93 (93.9)	0.218	1		
> 0	18 (8.9)	12 (11.7)	6 (6.1)		0.48 (0.17–1.35)		
Ecstasy use							
No	187 (92.6)	95 (92.2)	92 (92.9)	1.000	1		
Yes	15 (7.4)	8 (7.8)	7 (7.1)		0.90 (0.31–2.59)		
Ecstasy use time (in years)							
≤0	196 (97.0)	100 (97.1)	96 (97.0)	1.000	1		
> 0	6 (3.0)	3 (2.9)	3 (3.0)		1.04 (0.20–5.28)		
Ecstasy amount (tablets/day)							
≤0	187 (92.6)	95 (92.2)	92 (92.9)	1.000	1		
> 0	15 (7.4)	8 (7.8)	7 (7.1)		0.90 (0.31–2.59)		
Oxy use							
No	186 (92.1)	94 (91.3)	92 (92.9)	0.796	1		
Yes	16 (7.9)	9 (8.7)	7 (7.1)		0.79 (0.28–2.22)		
Oxy use time (in years							
≤0	197 (97.5)	102 (99.0)	95 (96.0)	0.205	1		
> 0	5 (2.5)	1 (1.0)	4 (4.0)		0.79 (0.47–39.11)		
Oxy amount (stones/day)							
≤0	186 (92.1)	94 (91.3)	92 (92.9)	0.796	1		
> 0	16 (7.9)	9 (8.7)	7 (7.1)		0.79 (0.28–2.22)		

Bold values are statistically significant (*P*<0.05) //

* Chi-square test

** Logistic regression and Wald's test

In the bivariate analysis, there was a statistically significant difference (*P*<0.05) and the worse impacts were reported by non-whites, those who reported feeling metallic taste, tooth mobility, smokers, who consume cigarettes for over 15 yr, who consume more than 3 g of crack a day, who have DMFT>10, and number of teeth ≤ 27. In the multivariate analysis, the worse impacts were reported, without loss of significance, by the same variables of the bivariate analysis (*P*<0.05), except for ethnicity (*P*=0.207).

## Discussion

This study used the OHIP-14 to assess the self-perception of DU regarding their oral health and associated variables, through the lack of findings in the literature for this purpose (9,16). The instrument having been widely used to evaluate the self-perception of oral health related to quality of life worldwide (21). It is presented as the main advantage for being of easy and fast application because it is a short-form with 14 questions but keeps the same goals as the original version containing 49 questions (17).

The OHIP-14 was used in a study including individuals addicted to alcohol and/or drugs that were being treated at a center for specialized dentistry in Amsterdam. The mean of the OHIP-score was 40.6. In the group studied, the poor oral health has a substantial impact on daily functioning (16). In contrast, in the present study, the impact was considered low. This low impact was also shown in studies with young populations (22). In addition, young people often have better opinions about their oral health and fewer cavities (23).

One of the reasons that can corroborate for this outcome is that the OHIP-14 questionnaire consists of questions with severe impacts and there are few individuals who answer the most serious dimensions (13).

The quality of life did not interfere with self-image and self-esteem in 100 polydrug users, male, with a mean age of 43 yr, in a Brazilian study (24). Oral health was considered good in 57.0% of the sample and 39.0% judged to be similar to the rest of the population, despite being measured by the World Health Organization Quality of Life (WHOQOL) (24).

The consequences of regular use of drugs for oral health are diverse (4–8). Therefore, some associated variables demonstrated impact on the quality of life for DU in the present study, corroborating previous findings (9).

Other results indicate mean ages close to those found here (2,10,25). It is observed in Brazil, where the profile of DU is similar to the data found in this study, i.e., young adults prevail (age 30 yr) (3), single, with low education (3), unemployed, from dysfunctional families and with risky sexual behavior (26).

There is indication in the literature that most of the DU have had employment until the time of admission (24), in agreement with the data found here.

The prevalent ethnic group in this study was white (75.0%), unlike the study where non-whites were predominant users (3). Non-white ethnic DU has two times more chances to have poorer self-perceived oral health. The results corroborate the literature findings, which indicate that there are greater account records of oral symptoms among non-whites compared to whites (27).

Higher education percentages have been demonstrated in other studies, in which 56.0% completed primary school (24), and 75.0% high school (25), differing from the present study with about 45.0% with low education and 33.0% with medium education. A Brazilian study also reported a low proportion of users with higher education (22). Although school surveys reveal problems associated with drug use (28), it is important for implementing preventive programs since the initial levels of schooling.

Despite the lack of statistical significance, the worst oral health impacts were reported in the present study by DU with medium and low education. This shows an inverse relationship between education and the perception of the impact, thus the lower education behaves as a factor that favors the reporting problems associated with poor oral health (23,29).

The major causes of tooth loss in the population are caries and periodontal disease, according to the last Brazilian National Survey (30). Thus, edentulous becomes one of the most impacting factors on quality of life, because it is related to function and nutrition (29). Additionally, advanced caries promotes discomfort, pain, infections that cause poor diet and weight loss (31).

DU has more dental problems, higher DMFT than the rest of the population (5–8), poor oral hygiene (4), and do not usually make regular visits to the dentist (10). Although the majority of respondents have confirmed the visit to the dentist in the last six months and brushing their teeth ≥ 3 times/day, the mean DMFT was greater than 10% to 43.0% of the sample. This relatively high value may result from deficient oral hygiene, since 76.7% reported not to floss and marijuana use (68.3%), which causes hyposalivation, lower frequencies of tooth brushing and dental control visits, in addition to the irregular consumption of cariogenic foods (32). This suggests lack of knowledge of oral hygiene techniques or even harm caused by the consumption of the drug. The DU with DMFT> 10 is 2.2 times more likely to poorer self-perceived oral health in this study. Authors have been observed that the DMFT index is not highly correlated with the self-assessment (33).

The tooth loss is commonly found in DU (6), especially due to caries and periodontal disease (6,25,34). In the present study, 2.7 teeth were missed due to caries, and 2.8 for other reasons. The mean number of missing teeth, reported in a study conducted in the United States, was 6 in 29.0% of DU (35). Here it was observed a reduced number of teeth in mouth and this fact increases the worse self-perception oral health, such as already observed (36). Age and tooth loss are closely related but have independent effects on quality of life in oral health. Tooth loss linked to aging presents the most negative impacts, whereas aging independently, resulting in less impact on the elderly (37). Another factor that may influence the oral health of DU is the time of dependency since the greater this is, the worse is the other (10).

In this study, the largest amount of crack was associated with worse outcomes. 41.1% of DU smoked an average of 3 g/day crack, considering that stone weighs around 0.24 g (37), they smoked 20 stones of *crack*/day for about seven years. This consumption was higher than that found in the Brazilian capitals whose average is 16 stones/day and lower than the eight years’ time consumption reported by the Ministry of Health (3).

The feeling of metallic taste that impacted oral health was reported by respondents, related to the presence of concomitant problems such as dental caries, periodontitis and the side effects of some drugs, which can change the taste (dysgeusia) (38). The DU may have periodontitis, but this was not assessed, and this is a limitation.

## Conclusion

The worse self-perceived oral health in drug users was associated with sociodemographic, behavioral and unfavorable habits, suggesting the need for public policies aimed at this population.
